# Association of gestational age with MRI-based biometrics of brain development in fetuses

**DOI:** 10.1186/s12880-020-00525-9

**Published:** 2020-11-25

**Authors:** Yuequan Shi, Yunjing Xue, Chunxia Chen, Kaiwu Lin, Zuofu Zhou

**Affiliations:** 1 Department of Radiology, Fujian Maternity and Child Health Hospital, Fuzhou, Fujian China; 2 Department of Radiology, Fujian Medical University Union Hospital, Fuzhou, Fujian China

**Keywords:** Magnetic resonance imaging, Fetal brain maturity, Gestational age

## Abstract

**Background:**

Reported date of last menstrual period and ultrasonography measurements are the most commonly used methods for determining gestational age in antenatal life. However, the mother cannot always determine the last menstrual period with certainty, and ultrasonography measurements are accurate only in the first trimester. We aimed to assess the ability of various biometric measurements on magnetic resonance imaging (MRI) in determining the accurate gestational age of an individual fetus in the second half of gestation.

**Methods:**

We used MRI to scan a total of 637 fetuses ranging in age from 22 to 40 gestational weeks. We evaluated 9 standard fetal 2D biometric parameters, and regression models were fitted to assess normal fetal brain development. A stepwise linear regression model was constructed to predict gestational age, and measurement accuracy was determined in a held-out, unseen test sample (n = 49).

**Results:**

A second-order polynomial regression model was found to be the best descriptor of biometric measures including brain bi-parietal diameter, head circumference, and fronto-occipital diameter in relation to normal fetal growth. Normal fetuses showed divergent growth patterns for the cerebrum and cerebellum, where the cerebrum undergoes rapid growth in the second trimester, while the cerebellum undergoes rapid growth in the third trimester. Moreover, a linear model based on biometrics of brain bi-parietal diameter, length of the corpus callosum, vermis area, transverse cerebellar diameter, and cerebellar area accurately predicted gestational age in the second and third trimesters (cross-validation *R*^*2*^ = 0.822, *p* < 0.001).

**Conclusions:**

These results support the use of MRI biometry charts to improve MRI evaluation of fetal growth and suggest that MRI biometry measurements offer a potential estimation model of fetal gestational age in the second half of gestation, which is vital to any assessment of pregnancy, fetal development, and neonatal care.

## Background

Accurate gestational dating is essential to any assessment of pregnancy, fetal development, and neonatal care. Before sonography, obstetricians routinely rely on the use of the last menstrual period (LMP) for dating gestational age (GA) in antenatal life [1, 2]. However, it has been reported that 20–40% of women cannot determine their last menstrual period with certainty due to various reasons such as late ovulation, bleeding or spotting during early pregnancy, erroneous recall, and pregnancy following the use of oral contraceptives [2–4]. The inaccuracy of the LMP has propelled the widespread use of linear biometric measurements of fetal sonography in utero as a more accurate method of assessing or confirming fetal gestational age [4]. Methods for estimating gestational age based on the measurement of crown‐rump length (CRL) in the first trimester, and fetal biometric measurements such as the brain bi-parietal diameter, head circumference, femur length, and abdominal circumference in the last two trimesters [1, 5], were reported decades ago and are still used today [6].

Although sonographic assessment within the first trimester is recognized as the most accurate estimate of gestational age, it shows large variation in the second and third trimesters due to variability in organ size [1, 4]. According to previous studies, gestational age assessment by combining the aforementioned biometric data can achieve an accuracy of ± 7 to 10 days for the second trimester and ± 21 to 30 days for the third trimester [7]. Further evidence indicates that a cerebellar length measurement, the transcerebellar diameter, is an accurate predictor of gestational age in both singleton and twin pregnancies [8, 9], but requires good visualization of the cerebellum by specialized sonographers. In summary, estimations made by sonographic measurement are strongly affected by the inherent variability of organ size and the intrinsic signal properties of ultrasonography [10]. The inaccuracy of sonographic assessment has propelled the need to find different approaches that can be used to accurately determine gestational age.

Magnetic resonance imaging (MRI) is being increasingly recognized as a powerful adjunct to ultrasonography in the evaluation of the fetal brain, as it provides high resolution, soft-tissue contrast and visibility of the whole brain independent of fetal presentation [11–14]. The high-resolution and rapid scanning time of MRI are advantageous for identifying anatomical structures and their accurate measurement, demonstrating an improvement in diagnostic accuracy of 23% when gestational age is between 18 and 24 weeks, and 29% at over 24 weeks, compared to ultrasound [15, 16]*.* Several MRI studies have shown that 2D biometric measurements and 3D volume can be used to characterize growth patterns and detect abnormalities in the fetus [17–20]. Moreover, a previous study demonstrated that a single whole-brain cortical folding measurement from MRI and simple linear regression can be used to accurately and reliably predict gestational age and brain maturity for 33 healthy fetuses in the third trimester [10]. However, the study sample size is relatively small and the time window is limited, warranting further confirmation of these findings.

Before we can begin to assess the usefulness of biometry measurements from MRI in determining fetal gestational age, better understanding of normal development of biometric markers in the fetus is essential. Although normative biometry data of ultrasound and MRI are putatively considered statistically equivalent [21], normative reference data established by sonography are not necessarily applicable to MRI due to differences in technique, imaging physics, and resolution [22, 23]. Hence, we sought to provide direct experimental evidence of fetal brain growth evaluation based on MRI biometrics in a large scale population. Specifically, we first investigated the relationship between MRI-based linear biometric data and brain development in 637 normal fetuses in a wide range of gestation age, from 22 to 40 weeks, which were screened from a large local database. Next, we were interested in whether there was a sex-specific difference in MRI-based biometric measures. Finally, we aimed to establish an MRI-based biometric predictive model to determine the accurate gestational age of an individual fetus.

## Methods

### Participants

From a database of 3251 fetal MRI scans that were performed at Fujian Maternity and Child Health Hospital between May 2012 and October 2017, we screened 896 (referred to as discovery dataset) prenatal MRI brain scans of fetuses that fulfilled the following criteria: (1) participants with a singleton pregnancy of > 14 weeks’ gestation which was determined with consistent estimation both by last menstrual period and ultrasonography dating (termed ‘measured GA’ here); (2) no history of exposure to risk factors or drug abuse during pregnancy; (3) no abnormality in structural brain anatomy in MRI. Exclusion criteria were: (1) delivery complications, congenital malformations or maternal infection, chromosomal abnormality, inadequate MRI image quality; (2) claustrophobia or contraindications to MRI. An independent held-out cohort comprising of 65 (referred to as validation dataset) normal fetuses aged from 23–38 weeks of gestation with MRI examination conducted between November 2017 and January 2018 was included as a validation dataset. This study was approved by the Institutional Review Board of Fujian Maternity and Child Health Hospital, and written informed consent for participation was obtained from all participants.

### Image acquisition

Fetal MRI data were collected using a 1.5 T Signa (General Electric Medical Systems, Milwaukee, WI) whole-body MRI scanner with an 8-channel phased array body coil. The mother was positioned feet-first into the scanner without sedation, and was instructed to stay as relaxed as possible to reduce spontaneous motion of the fetus. A rapid localizer was acquired using a three-plane single-shot fast spin echo (SS-FSE) sequence with the following scanning parameters: repetition time (TR) = minimal (39.8 ms), echo time (TE) = 80 ms, field of view (FOV) = 48 × 48 cm, matrix = 288 x 128, scan time = 6 s. From this localizer, images in all three orthogonal directions (axial, sagittal, coronal) were acquired with both 2D Fast imaging employing steady-state acquisition (FIESTA) and SS-FSE. The parameters used for all 2D FIESTA scans were: TR = 3.9 ms, TE = minimal (1.4 ms), FOV = 42 × 42 cm, flip angle (FA) = 75$^\circ$, matrix = 256 x 320, slice thickness was 3 to 5 mm with 1 mm interslice gap, NEX = 1, scan time = 25 s. Scanning parameters for SS-FSE were as follows: TR = 3000 ms, TE = 68 ms, FA = 80$^\circ$, FOV = 42 × 42 cm, matrix = 384 x 320, slice thickness was 3 to 5 mm with 1 mm interslice gap, NEX = 1, scan time = 47 s. The total scan time for the mother was 3 to 4 minutes.

### Image processing and analysis

**Fig. 1:**
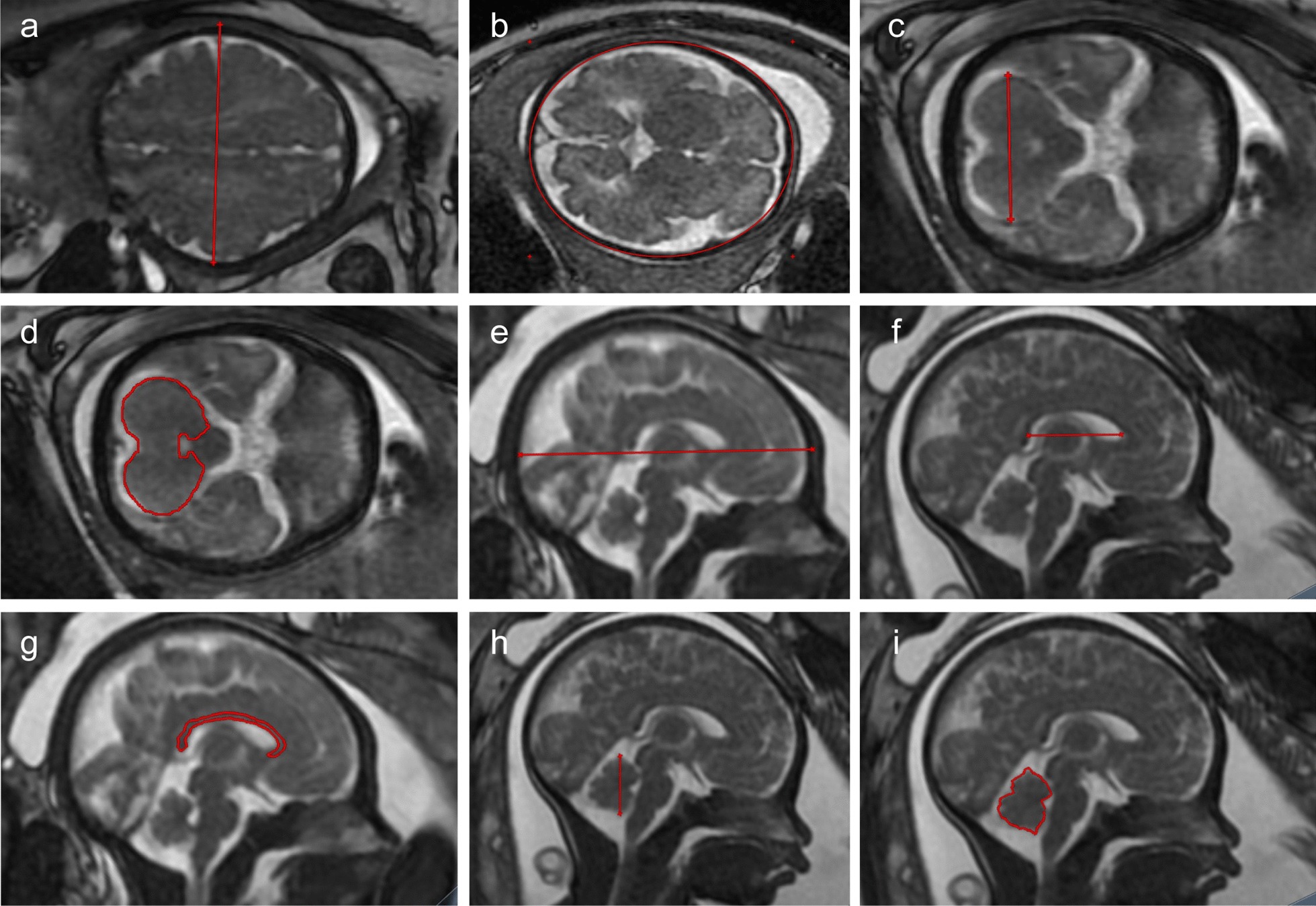
Illustration of magnetic resonance imaging slice showing measurements of: **a** brain biparietal diameter; **b** head circumference; **c** transverse cerebellar diameter; **d** cerebellar area; **e** fronto-occipital diameter; **f** length of the corpus callosum; **g** corpus callosum area; **h** vermis height; **i** vermis area

Acquired MRI images were stored in the picture archiving and communication system (PACS). Both MRI data and fetal anatomy were evaluated by two experienced radiologists. Linear biometric measurements were made on 2D MRI images including: brain bi-parietal diameter (BPD), head circumference (HC), transverse cerebellar diameter (TCD), cerebellar area (CA), fronto-occipital diameter (FOD), length of the corpus callosum (LCC), corpus callosum area (CCA), and vermis height (VH) and area (VA), all of which were defined as follows:

1. BPD, defined as the widest diameter of the fetal skull measured in a transverse plane using the “outer edge to inner edge’’ technique (Fig. 1a).
2. HC, defined as the maximum circumference of the fetal skull measured at the level of the transverse plane that traverses the thalami (Fig. 1b).
3. TCD, defined as the largest cerebellar diameter in the transverse plane that traverses the cerebellar peduncles (Fig. 1c).
4. CA, defined as the maximum area of cerebellum in the transverse plane that traverses the cerebellar peduncles (Fig. 1d).
5. FOD, defined as the longest distance between the extreme point of the frontal and occipital lobes measured in a midline sagittal plane (Fig. 1e).
6. LCC, defined as the length from anterior tip of the genu to the posterior tip of the splenium measured on the midline sagittal plane (Fig. 1f).
7. CCA, defined as the maximum area of the corpus callosum measured on the midline sagittal plane (Fig. 1g).
8. VH, defined as the maximum superior–inferior length of the vermis in the midsagittal plane (Fig. 1h).
9. VA, defined as the maximum area of the vermis in the midsagittal plane (Fig. 1i).

Note that sub-millimeter measurements were rounded with regard to the voxel size of MRI images even though they were feasible in the post-processing console.

### Statistical analysis

Statistical analysis was performed using Matlab (Mathworks Inc. version 2014a). Two experienced radiologists were involved in assessing the linear biometric measurements. The BPD was measured for 30 randomly selected fetuses two times by each radiologist, who was blind to the fetus gestational age. Intra- and inter-rater reliability were evaluated within and across radiologists using Pearson’s correlation.

A non-parametric Wilcoxon rank-sum test was performed to estimate difference in GA distribution and biometric measurements between male and female fetuses. The relationship between MRI-based linear biometric data and GA was conducted in the discovery dataset. Specifically, linear and quadratic regression models were used to assess the relationship between gestational age and biometric measurements. Goodness-of-fit was compared using adjusted R^2^ for the regression models. Finally, in the discovery dataset, a stepwise linear regression was applied to model the relationship between the dependent variable (gestational age) and independent variables (fetal biometric measurements), which iteratively determined a combination of biometric measurements that were linearly linked to GA. At each iteration, one biometric measurement was added or removed from the model for better fitting. Afterwards, the determined model was applied to predict the GA for unseen fetuses in the held-out validation cohort to assess the generalizability of the established model. The Pearson correlation coefficient and cross-validation *r*^2^ [24] between the predicted and measured GA were used to assess the predictive performance.

## Results

### Participant demographic information

Biometric measurements were successfully acquired from 637 (male = 357, mean $\pm$ SD GA (week) = 31.39 $\pm { }$ 3.91; female = 280, mean $\pm { }$ SD GA (week) = 31.88 $\pm { }$ 3.61) (Table 1) fetal MRI images in the cohort of 896 fetuses aged 22–40 gestational weeks in the discovery dataset. There was no significant difference in gestational age between male and female (*Z* = − 1.607, *p* = 0.108). In the held-out validation dataset, biometric measurements were successfully acquired from 49 of 65 fetuses (male = 20, mean $\pm { }$ SD GA (week) = 31.95 $\pm { }$ 3.98, range = 23–38; female = 29, mean $\pm { }$ SD GA (week) = 32.83 $\pm { }$ 2.35, range = 28—38) (Table 1).

**Table 1:** Age and sex distribution in discovery and validation datasets

GA (week)	Discovery dataset		Validation dataset	
	Male (N)	Female (N)	Male (N)	Female (N)
22	5	4	0	0
23	8	4	1	0
24	10	7	0	0
25	11	5	0	0
26	11	5	0	0
27	13	8	3	0
28	23	9	1	2
29	23	20	0	1
30	24	16	1	1
31	31	24	0	2
32	53	46	4	7
33	37	49	4	4
34	41	33	2	7
35	21	13	0	2
36	13	11	1	1
37	11	10	1	1
38	13	8	2	1
39	7	6	0	0
40	2	2	0	0
Total	357	280	20	29

### Intra-rater and inter-rater reliability

Pearson correlation coefficients showed that the intra-rater reliability was *r* = 0.996 (*p* < 0.001), *r* = 0.988 (*p* < 0.001) for the two radiologists, respectively. Their inter-reliability was *r* = 0.961 (*p* < 0.001).

### Sex effect for biometric measurements

There was no significant sex effect for TCD (*Z* = − 0.372, *p* = 0.710), CA (*Z* = − 0.770, *p* = 0.442), LCC (*Z* = − 0.140, *p* = 0.889), CCA (*Z* = − 0.106, *p* = 0.916), VA (*Z* = − 0.953, *p* = 0.341), and VH (*Z* = − 1.478, *p* = 0.139). By contrast, there was a statistically significant sex effect for BPD (*Z* = 1.985, *p* = 0.004), FOD (*Z* = 2.343, *p* = 0.019), and HC (*Z* = 2.547, *p* = 0.011) (Additional file 1: Table S1). We conducted a post-hoc analysis to determine whether there were significant differences in the BPD, FOD, and HC between male and female fetuses at each GA period. We observed that male fetuses had significantly larger BPD (Additional file 1: Table S2), FOD (Additional file 1: Table S3), and HC (Additional file 1: Table S4) than females from 31 weeks onward, whereas the difference became statistically insignificant from 35 weeks onward. Note that differences in BPD, FOD and HC were rather small such that they may not be considered clinically meaningful.

### Relationship between biometric measurements and GA

**Fig. 2:**
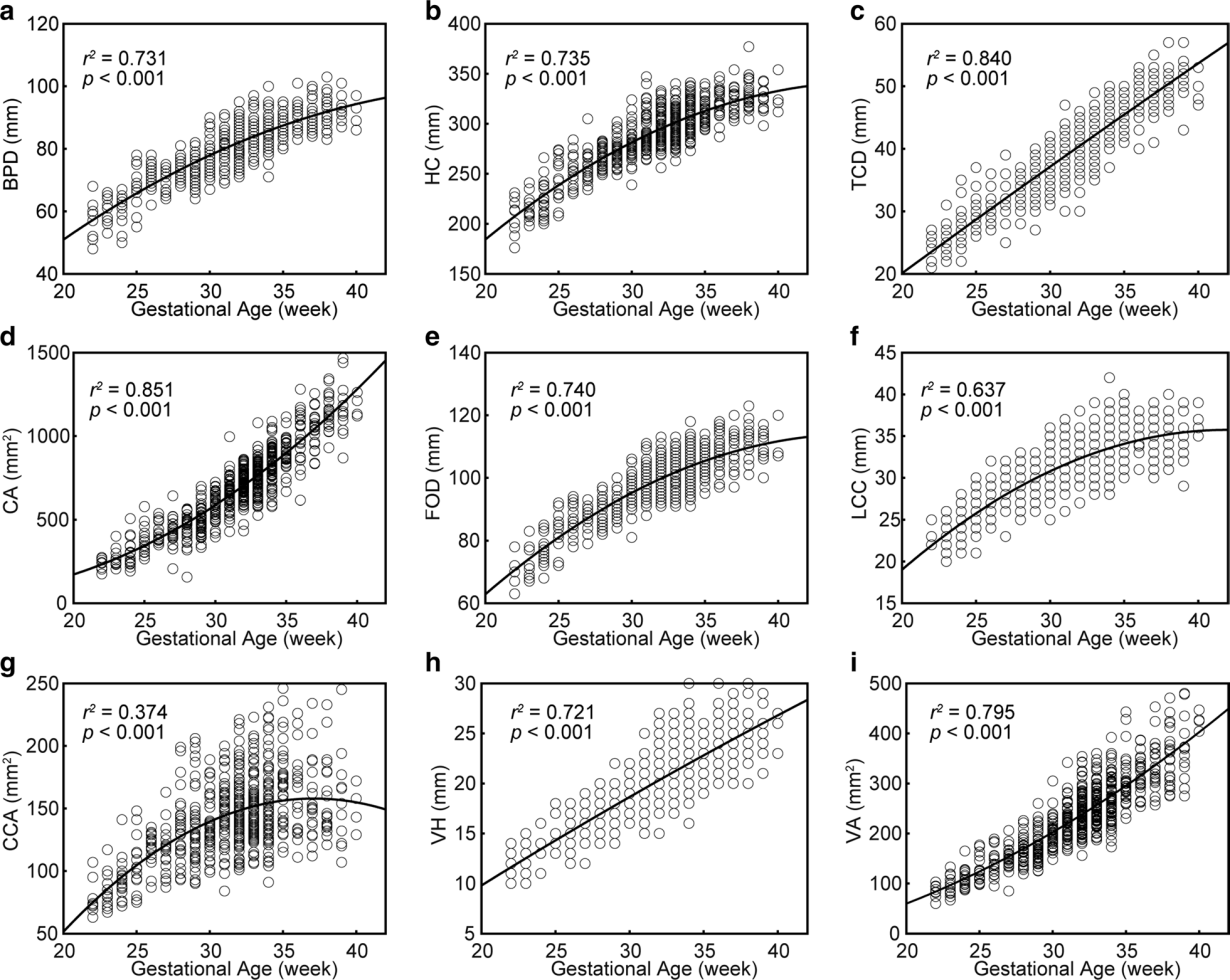
Best fit models for the relationship between the 9 chosen biometric measurements and gestational age in the discovery dataset. **a** BPD, the brain biparietal diameter; **b** HC, head circumference; **c** TCD, transverse cerebellar diameter; **d** CA, cerebellar area; **e** FOD, fronto-occipital length; **f** LCC, length of the corpus callosum; **g** CCA, corpus callosum area; **h** VH, vermis height; **i** VA, vermis area

As shown in Fig. 2, the quadratic relation was the best-fitting model for the positive correlation between all biometric measurements and GA. The nonlinear pattern is in keeping with the following observations: 1) Rapid nonlinear growth rate of the BPD (Fig. 2a), HC (Fig. 2b), FOD (Fig. 2e), LCC (Fig. 2f), and CCA (Fig. 2g) in the second trimester, followed by relatively slower growth rate in the third, and 2) In contrast, slower growth of the CA (Fig. 2d) and VA (Fig. 2i) in the second trimester, followed by rapid growth in the third trimester. Note that although the regression looks linear for TCD and VH, the second order polynomial model better fit the TCD (Fig. 2c, adjusted R^2^ = 0.840) and VH (Fig. 2h, adjusted R^2^ = 0.721) than the linear model (TCD: adjusted R^2^ = 0.838, VH: adjusted R^2^ = 0.694).

Finally, the stepwise linear regression revealed that a linear model on the basis of biometric measurements including BPD, LCC, VA, CA, and TCD achieved a correlation of *r* = 0.935 (*p* < 0.001) between the predicted and measured GA in the discovery dataset. The linear model was expressed as follows:

$${\text{GA}} = 11.413 + 0.062 \times {\text{BPD}} + 0.118 \times {\text{LCC}} + 0.008 \times {\text{VA}} + 0.004 \times {\text{CA}} + 0.173 \times {\text{TCD}}$$

**Fig. 3:**
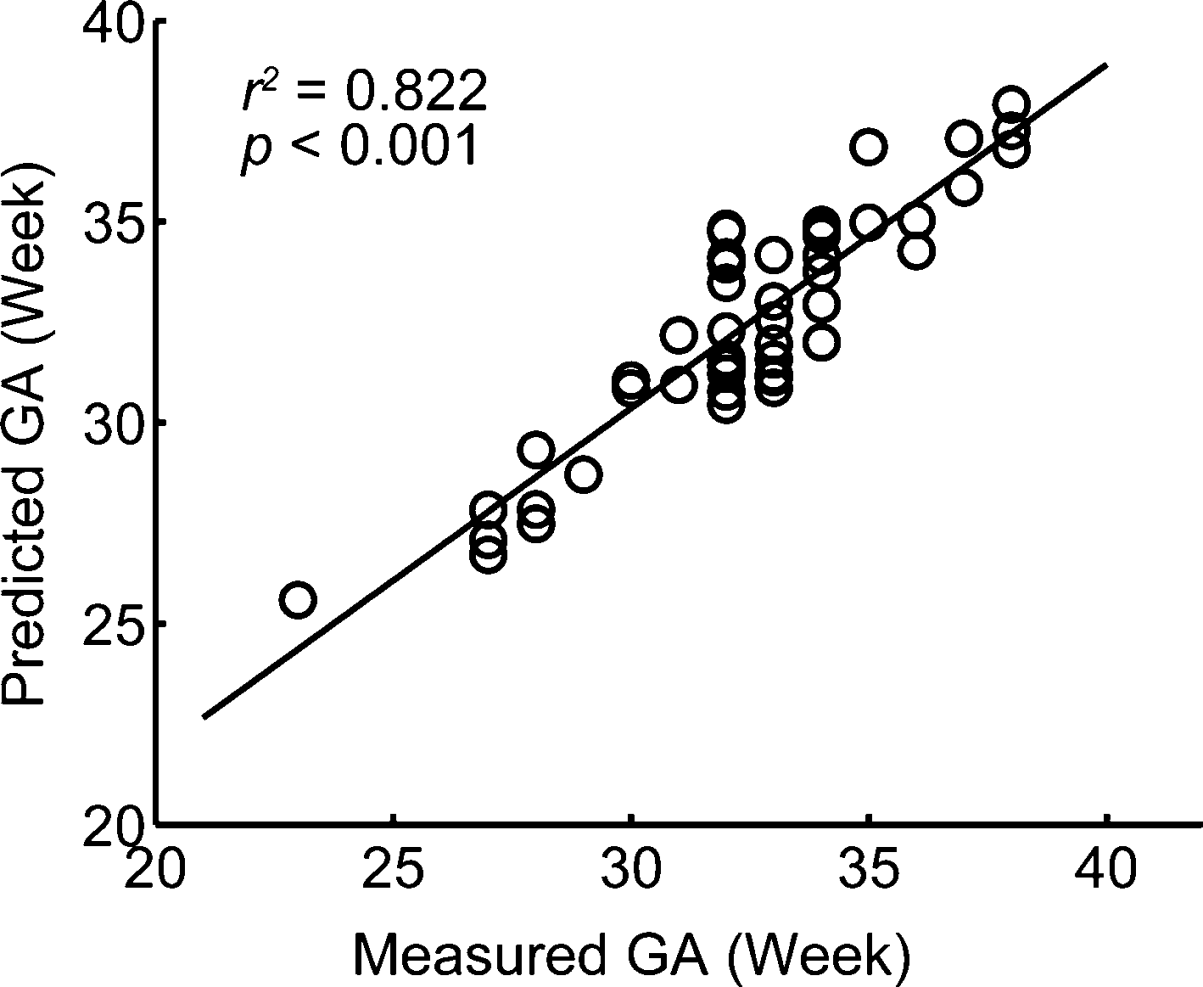
Predicted GA was significantly correlated with measured GA in the held-out validation dataset using the built model based on biometric measurements, including brain biparietal diameter, length of the corpus callosum, vermis area, cerebellar area, and transverse cerebellar diameter. *GA* gestational age

The built linear model was then applied to predict individualized GA for unseen fetuses in the holdout validation dataset to evaluate generalizability. There was a significant positive correlation between the predicted and measured GA in held-out validation dataset (*r* = 0.907, cross-validation *R*^*2*^ = 0.822, *p* < 0.001) (Fig. 3). This validates the generalizability of the identified biometric measurements, which may be broadly applicable as predictors of GA for new fetuses.

## Discussion

The present study delineated the growth trajectory for linear biometric measurements and established an MRI-based biometric predictive model to accurately determine gestation age in a large cohort of normal fetuses (n = 637) from 22 to 40 gestational weeks. Specifically, the quadratic growth pattern was demonstrated as the best-fitting model describing the relationship between these measurements and gestational age. Moreover, a linear model based on the brain bi-parietal diameter, length of the corpus callosum, vermis area, cerebellar area, and transverse cerebellar diameter was able to effectively predict the gestational age for unseen fetuses. Collectively, these findings underscore the specific developmental patterns of brain biometrics during the second and third trimesters, thereby providing additional vital information to aid in prenatal assessment.

We observed that an increase in these biometric measurements along with gestational age followed a polynomial regression model, in agreement with previous regression analysis of MRI [20, 21, 25, 26] and ultrasound measurement data [27–29]. Evidently, healthy fetuses exhibited different growth patterns for cerebrum and cerebellum during pregnancy. The basic morphology of the cerebellum, which results from neuronal proliferation, directional migration, and differentiation, is formed around the 20th week of gestation [30]. Until term, growth of the cerebellum is dominated by the inward migration from the external to internal granular layers of the cortex, followed by outgrowth of fibers that make up the cerebellar cortical circuits [31]. This corresponds to the cerebellum undergoing faster increases in volume and surface foliation than other cerebral structures [32, 33], as may constitute relatively faster growth of the cerebellum. This observation is consistent with previous reports of 3D volumetric MRI studies, thus highlighting the exuberant and accelerated period for cerebellar development occurring in the third trimester. During this critical period, the remarkably rapid growth of cerebellum takes a distinctive lead among other cerebrum structures [32, 34–36]. Recognizing the in utero fetal brain heterochronic development may enhance detection of cognitive and neuropsychiatric diseases that otherwise would present later in childhood or young adulthood.

Compared to female fetuses, males have statistical significantly larger brain bi-parietal diameter, fronto-occipital diameter, and head circumference, as reiterates the extent of sex-related differences in brain development [25, 37–39]. In general, differences of 1.0 mm in brain bi-parietal diameter, 2.0 mm in fronto-occipital diameter, and 0.5 mm in head circumference between sexes were identified at quite a small scale, all of which might not have clear clinical meanings for prenatal evaluation [20, 25, 37–40].

Establishing an accurate gestational age and estimated delivery date is the most important step in the management of any pregnancy. Accurate knowledge of gestational age is vital for timing of appropriate obstetric care; scheduling and interpretation of certain antepartum tests; determining the appropriateness of fetal growth; and designing interventions to prevent preterm births, postterm births, and related morbidities [41]. As sonographic measure of the bi-parietal diameter has been extensively studied and well reproduced [42], it has been recommended as a strong marker for dating. Caution is warranted since faithful accuracy for this measurement has been demonstrated predominantly in the first trimester. In contrast, transverse cerebellar diameter is not likely to be affected by factors affecting fetal growth, which has been considered as an alternative accurate predictor in estimation of gestational age for the second and third trimesters, but requires good visualization of the cerebellum [8, 9, 43]. As a matter of fact, it has been demonstrated that measurement of the vermis together with cerebellar area could improve the predictive accuracy of gestational age relative to single measurement [44]. The present study further demonstrates that a linear combination of brain bi-parietal diameter, the length of the corpus callosum, the vermis area, the cerebellar area, and the transverse cerebellar diameter was able to accurately predict the gestational age in normal fetuses. Importantly, MRI is unhampered by an ongoing ossification of the fetal skull, the increased physical size of the woman, or the descent of the fetal head into the maternal pelvis to achieve more accurate biometry measurements in the second and third trimester of pregnancy [16]. In summary, multiple MRI-based characteristics of brain development should be considered together to accurately evaluate fetal brain maturity in the last two trimesters of pregnancy.

The present study should be interpreted in view of its limitations. The sample size of each gestational age period in the held-out validation dataset is relatively small, and replication to validate the present predictive model with data from larger populations and settings will be critical to extend the current use of this MRI-based biometry measurements predictive model to clinical application scenarios. Since fetal growth is influenced by each mother's previous gestational history and body condition and composition, future studies should consider mothers’ demographics as variables or covariables in models that might be useful to improve gestational dating precision.

## Conclusion

In summary, we demonstrated nonlinear development trajectories of MRI-based biometric brain measures across a wide range of gestational ages in a large population, and distinct patterns of regional growth for the cerebrum and cerebellum. Importantly, the availability of in utero MRI-based biometric measures to accurately predict the gestational age of a fetus in the second and third trimester will offer a novel way to determine the appropriate intervals for prenatal care visits, as well as the timing of certain interventions. Our findings enhance the present understanding of brain development during fetal growth, which may help improve clinical neonatal care in the future.

## Supplementary information

**Additional file 1**. Sex effect for biometric measurements.

## Data Availability

The datasets used and/or analyzed during the current study are available from the corresponding author on reasonable request.

## Abbreviations

MRI: Magnetic resonance imaging
GA: Gestational age
BPD: The brain biparietal diameter
HC: Head circumference
TCD: Transverse cerebellar diameter
CA: Cerebellar area
FOD: Fronto-occipital length
LCC: Length of the corpus callosum
CCA: Corpus callosum area
VH: Vermis height
VA: Vermis area
PACS: Picture archiving and communication system
