# Supplementary material for: Association of gestational age with MRI-based biometrics of brain development in fetuses
Source: BMC Med Imaging. 2020 Nov 25;20:125. doi: 10.1186/s12880-020-00525-9 (PMC7689975; doi:10.1186/s12880-020-00525-9)
Supplement: Supplementary file 1 — Additional file 1. Sex effect for biometric measurements. [file 12880_2020_525_MOESM1_ESM.docx]

**Supplementary material for**

**Association of gestational age with MRI-based biometrics of brain development in fetuses**

Yuequan Shi^1^, Yunjing Xue^2^, Chunxia Chen^1^, Kaiwu Lin^1^, Zuofu Zhou^1*^

^*^**Corresponding author:**

Zuofu Zhou, M.D, Ph.D

Department of Radiology, Fujian Maternity and Child Health Hospital, China

E-mail: fzzzf1968@163.com

Tel: 86-0591-86329381

## Table S1. Sex effect for biometric measurements. Data were presented as median [range]. Bold font denotes significant *p*< 0.05 using Non-parameter Wilcoxon rank-sum test.

| Measurements | Male | Female | *Z* | *P* |
| --- | --- | --- | --- | --- |
| **BPD (mm)** | **82.00 [50.00, 100.00]** | **81.00 [48.00, 103.00]** | **1.985** | **0.047** |
| **HC (mm)** | **100.00 [67.00, 123.00]** | **98.00 [63.00, 120.00]** | **2.547** | **0.011** |
| TCD (mm) | 297.00 [201.00, 377.00] | 288.00 [176.00, 354.00] | -0.372 | 0.71 |
| CA (mm^2^) | 32.00 [20.00, 42.00] | 32.00 [20.00, 39.00] | -0.77 | 0.442 |
| **FOD (mm)** | **139.00 [72.00, 245.00]** | **138.50 [63.00, 246.00]** | **2.343** | **0.019** |
| LCC (mm) | 230.00 [67.00, 478.00] | 236.00 [60.00, 480.00] | -0.14 | 0.889 |
| CCA (mm^2^) | 20.00 [10.00, 30.00] | 20.00 [10.00, 30.00] | -0.106 | 0.916 |
| VH (mm) | 695.00 [156.00, 1343.00] | 703.50 [175.00, 1466.00] | -1.478 | 0.139 |
| VA (mm^2^) | 40.00 [22.00, 57.00] | 40.00 [21.00, 57.00] | -0.953 | 0.341 |

Abbreviations: BPD, the brain biparietal diameter; HC, head circumference; TCD, transverse cerebellar diameter; CA, cerebellar area; FOD, fronto-occipital length; LCC, length of the corpus callosum; CCA, corpus callosum area; VH, vermis height; VA, vermis area.

## Table S2. Sex effect for the BPD at each GA. Data were presented as median [range]. Bold font denotes significant *p*< 0.05 using Non-parameter Wilcoxon rank-sum test.

| GA (Week) | Male | Female | *Z* | *P* |
| --- | --- | --- | --- | --- |
| 22 | 59.00 [57.00, 68.00] | 51.50 [48.00, 60.00] | 1.599 | 0.11 |
| 23 | 61.50 [57.00, 66.00] | 56.00 [53.00, 64.00] | 1.707 | 0.088 |
| 24 | 62.00 [50.00, 67.00] | 61.00 [57.00, 65.00] | 0.295 | 0.768 |
| 25 | 67.00 [55.00, 78.00] | 66.00 [58.00, 78.00] | 0.456 | 0.649 |
| 26 | 71.00 [62.00, 78.00] | 73.00 [67.00, 76.00] | -0.454 | 0.649 |
| **27** | **69.00 [64.00, 75.00]** | **74.00 [67.00, 75.00]** | **-2.305** | **0.021** |
| 28 | 75.00 [69.00, 81.00] | 73.00 [68.00, 77.00] | 1.478 | 0.14 |
| **29** | **75.00 [68.00, 82.00]** | **72.25 [64.00, 82.00]** | **2.271** | **0.023** |
| 30 | 76.50 [70.00, 90.00] | 76.50 [67.00, 83.00] | 0.389 | 0.697 |
| **31** | **81.80 [69.00, 91.00]** | **78.00 [70.00, 87.00]** | **2.865** | **0.004** |
| **32** | **83.00 [73.00, 95.00]** | **80.00 [71.00, 88.00]** | **4.153** | **< 0.001** |
| **33** | **85.00 [80.00, 97.00]** | **83.00 [75.00, 95.00]** | **2.536** | **0.011** |
| **34** | **86.00 [75.00, 100.00]** | **84.00 [71.00, 93.00]** | **2.263** | **0.024** |
| 35 | 89.00 [81.00, 95.00] | 86.00 [80.00, 92.00] | 1.814 | 0.07 |
| 36 | 90.00 [83.00, 95.00] | 92.00 [86.00, 101.00] | -1.108 | 0.268 |
| 37 | 90.00 [85.00, 99.00] | 88.50 [84.00, 100.00] | 0.672 | 0.501 |
| 38 | 91.00 [83.00, 99.00] | 91.00 [85.00, 103.00] | 0.145 | 0.884 |
| 39 | 92.00 [87.00, 97.00] | 93.00 [89.00, 101.00] | -0.649 | 0.516 |
| 40 | 93.00 [89.00, 97.00] | 89.50 [86.00, 93.00] | 0.387 | 0.699 |

Abbreviations: GA, gestational age; BPD, the brain biparietal diameter.

## Table S3. Sex effect for the FOD at each GA. Data were presented as median [range]. Bold font denotes significant *p*< 0.05 using Non-parameter Wilcoxon rank-sum test.

| GA (Week) | Male | Female | *Z* | *P* |
| --- | --- | --- | --- | --- |
| 22 | 72.00 [70.00, 78.00] | 67.00 [63.00, 78.00] | 1.257 | 0.209 |
| 23 | 70.50 [67.00, 83.00] | 71.50 [68.00, 72.00] | 0 | 1 |
| 24 | 76.00 [68.00, 83.00] | 76.00 [72.00, 85.00] | -0.147 | 0.883 |
| 25 | 84.00 [73.00, 91.00] | 81.00 [72.00, 92.00] | 0.398 | 0.691 |
| 26 | 86.00 [78.00, 94.00] | 85.00 [78.00, 93.00] | 0.683 | 0.495 |
| 27 | 84.00 [79.00, 93.00] | 89.00 [86.00, 93.00] | -1.787 | 0.074 |
| 28 | 92.00 [83.00, 96.00] | 90.00 [82.00, 97.00] | 1.2 | 0.23 |
| **29** | **93.00 [88.00, 100.00]** | **90.50 [84.00, 100.00]** | **2.534** | **0.011** |
| 30 | 95.00 [87.00, 106.00] | 94.00 [81.00, 100.00] | 0.803 | 0.422 |
| **31** | **100.00 [91.00, 111.00]** | **95.50 [88.00, 102.00]** | **3.445** | **0.001** |
| **32** | **103.00 [91.00, 113.00]** | **98.00 [91.00, 104.00]** | **3.75** | **< 0.001** |
| **33** | **103.00 [91.00, 113.00]** | **100.00 [92.00, 111.00]** | **2.277** | **0.023** |
| **34** | **105.00 [91.00, 118.00]** | **101.00 [93.00, 114.00]** | **2.43** | **0.015** |
| **35** | **107.00 [95.00, 114.00]** | **103.00 [97.00, 109.00]** | **2.132** | **0.033** |
| 36 | 108.00 [97.00, 118.00] | 108.00 [101.00, 112.00] | 0.696 | 0.486 |
| **37** | **111.00 [106.00, 115.00]** | **103.00 [97.00, 120.00]** | **2.368** | **0.018** |
| 38 | 110.00 [104.00, 123.00] | 112.50 [101.00, 115.00] | -0.582 | 0.56 |
| 39 | 109.00 [100.00, 115.00] | 112.50 [106.00, 117.00] | -0.574 | 0.566 |
| 40 | 114.00 [108.00, 120.00] | 107.50 [107.00, 108.00] | 0.816 | 0.414 |

Abbreviations: GA, gestational age; FOD, fronto-occipital length.

## Table S4. Sex effect for the HC at each GA. Data were presented as median [range]. Bold font denotes significant *p*< 0.05 using Non-parameter Wilcoxon rank-sum test.

| GA (Week) | Male | Female | *Z* | *P* |
| --- | --- | --- | --- | --- |
| 22 | 214.00 [201.00, 227.00] | 192.00 [176.00, 231.00] | 1.102 | 0.27 |
| 23 | 214.00 [203.00, 241.00] | 208.50 [198.00, 224.00] | 1.106 | 0.269 |
| 24 | 215.50 [203.00, 266.00] | 225.00 [200.00, 252.00] | -0.439 | 0.661 |
| 25 | 243.00 [208.00, 274.00] | 236.00 [211.00, 273.00] | 0.227 | 0.82 |
| 26 | 256.00 [230.00, 284.00] | 245.00 [232.00, 267.00] | 1.361 | 0.173 |
| 27 | 251.00 [234.00, 276.00] | 266.00 [245.00, 305.00] | -1.632 | 0.103 |
| 28 | 265.00 [253.00, 283.00] | 266.00 [249.00, 282.00] | 0.545 | 0.586 |
| **29** | **272.00 [254.00, 302.00]** | **263.00 [249.00, 295.00]** | **2.254** | **0.024** |
| 30 | 280.00 [255.00, 324.00] | 277.00 [239.00, 290.00] | 1.008 | 0.313 |
| **31** | **290.00 [274.00, 347.00]** | **281.50 [264.00, 303.00]** | **3.294** | **0.001** |
| **32** | **306.00 [269.00, 333.00]** | **287.50 [256.00, 329.00]** | **4.959** | **< 0.001** |
| **33** | **305.00 [274.00, 340.00]** | **292.00 [263.00, 333.00]** | **2.788** | **0.005** |
| **34** | **313.00 [282.00, 341.00]** | **299.00 [273.00, 334.00]** | **3.443** | **0.001** |
| 35 | 314.00 [297.00, 340.00] | 312.00 [294.00, 331.00] | 0.692 | 0.489 |
| 36 | 322.00 [287.00, 347.00] | 328.00 [309.00, 343.00] | -0.261 | 0.794 |
| 37 | 323.00 [303.00, 354.00] | 312.00 [303.00, 354.00] | 0.847 | 0.397 |
| 38 | 327.00 [305.00, 377.00] | 328.00 [316.00, 349.00] | -0.362 | 0.717 |
| 39 | 324.00 [298.00, 343.00] | 328.50 [315.00, 342.00] | -0.788 | 0.431 |
| 40 | 337.50 [321.00, 354.00] | 318.00 [312.00, 324.00] | 0.387 | 0.699 |

Abbreviations: GA, gestational age; HC, head circumference.
